# Hereditary pancreatitis model by blastocyst complementation in mouse

**DOI:** 10.18632/oncotarget.27595

**Published:** 2020-06-02

**Authors:** Ayumu Asai, Masamitsu Konno, Koichi Kawamoto, Ayako Isotani, Masaki Mori, Hidetoshi Eguchi, Yuichiro Doki, Takahiro Arai, Hideshi Ishii

**Affiliations:** ^1^ Department of Frontier Science for Cancer and Chemotherapy, Graduate School of Medicine, Osaka University, Suita 565-0871 Japan; ^2^ Department of Medical Data Science, Graduate School of Medicine, Osaka University, Suita 565-0871 Japan; ^3^ Department of Gastroenterological Surgery, Graduate School of Medicine, Osaka University, Suita 565-0871 Japan; ^4^ Organ Developmental Engineering, Division of Biological Science, Graduate School of Science and Technology, Nara Institute of Science and Technology, Ikoma 630-0192 Japan; ^5^ Department of Surgery and Science, Graduate School of Medical Sciences, Kyushu University, Fukuoka 812-8582 Japan; ^6^ Unitech Co., Ltd., Kashiwa 277-0005 Japan

**Keywords:** blastocyst complementation, hereditary pancreatitis, disease-specific pluripotent stem cells, PRSS1

## Abstract

The application of pluripotent stem cells is expected to contribute to the elucidation of unknown mechanism of human diseases. However, *in vitro* induction of organ-specific cells, such as pancreas and liver, is still difficult and the reproduction of their disorders in a model has been unfeasible. To study the mechanism of human hereditary pancreatitis (HP), we here performed the blastocyst complementation (BC) method. In the BC method, mouse embryonic stem (ES) cells harboring CRISPR/CAS9-mediated mutations in the *Prss1* gene were injected into blastocysts with deficient *Pdx1* gene, which is a critical transcription factor in the development of pancreas. The results showed that trypsin was activated extremely in Prss1-mutant mice. This implied that the mouse phenotype mimics that of human HP and that the BC method was useful for the reproduction and study of pancreatic disorders. The present study opens the possibility of investigating uncharacterized human diseases by utilizing the BC method.

## INTRODUCTION

Pluripotent stem cells (PSCs)-applied technologies enable reprogramming of cells even harboring disease-specific germline mutations. Therefore, disease-specific PSCs are regarded as important tools to monitor disease processes, to explore drug screening and discovery, and to elucidate the pathophysiology of human diseases [1, 2]. Previous studies have used disease-specific PSCs from patients with hereditary disorders to clarify the pathogenesis of muscle dystrophy [3] and fibrodysplasia ossificans progressiva [4]. These studies were performed *in vitro*. Although reproduction of endocrine diseases such as pancreatic diseases were also studied [5, 6], there has been no enough model to conclude consistent results between *in vitro* and *in vivo*, presumably due to the difficulty of cell differentiation *in vitro* and the complexity of the organ structures [7–9]. In order to reproduce the diseases that are derived from tissues, in which induction of differentiation is difficult, it is undoubtedly necessary to improve the efficiency of differentiation induction and to reconstruct the complexity of tissues in a model.

A recent report indicated that a 3D human induced-pluripotent stem cells (iPSCs) engineered heart tissue was a useful tool for modeling *torsade de pointes*, which is a lethal arrhythmia that is often drug-induced; in particular, the 3D model provided details on the mechanisms underlying arrhythmia generation and on the means for drug discovery and safety tests [10]. Moreover, other studies have reported the usefulness of patient-derived iPSCs as a screening tool of biomarkers for Alzheimer's disease [11] and Behçet's disease [12]. The use of PSCs and animal model is expected to cause a breakthrough in the discovery of drugs that target rare genetic disorders, which accounts for approximately 7,000 diseases that affect millions of individuals in the United States [13]. Given that disease-specific PSCs are in the undifferentiated state, in which alterations in disease-causing gene sets are preserved [1, 2], a technology that induces differentiation of the disease-specific PSCs would be expect to reproduce the initial process of a disease.

The blastocyst complementation (BC) method has attracted an attention as a model that could reproduce human tissues and diseases in other animals and has been reported to be applicable especially for the construction of pancreatic tissues [14]. Mice with the knockout allele of the pancreatic and duodenal homeobox 1 (*Pdx1*) gene, which is a critical transcription factor that determines the fate of pancreatic endocrine and exocrine differentiation, have been reported to manifest with lethal phenotypes immediately after birth due to a defective formation of the pancreas, and injection of wild-type embryonal stem cells (ESCs) in a knockout blastocyst of the *Pdx1* gene rescued the mice completely [14, 15]. Similarly, reproduction of lungs by the BC method has recently been reported [16]. The BC method has been successful not only in mice, but also in pigs that are genetically closer to humans [17]. Moreover, the BC method was applied for pancreatic formation in an intercross species condition (i. e., rats to mice or vice versa) [15]. Thus, this method would be able to apply to human in the future.

Although the BC method using disease-specific PSCs had been suggested to reproduce hereditary diseases *in vivo*, its application on the study of hereditary diseases has been reported by only a few. In the present study, thus, we aimed to reproduce hereditary pancreatitis (HP) using the BC method.

HP is a relatively rare disorder of the pancreas [18–20] and was first reported by Comfort and Steinberg in 1952 [21]. HP is associated with pancreatic inflammation and had been attributed to genetic causes, such as mutation of the cationic trypsinogen or protease serine 1 (*PRSS1*) gene in the long arm of chromosome 7q35, which was the first isolated gene responsible for HP [22]. In general, translation of the *PRSS1* leads to the production of the precursor of trypsin, which is cleaved off from the region of its signal peptide and the trypsinogen-activating peptide, resulting in the activation of trypsin; on the other hand, cleavage of activated trypsin causes inactivation. Substitution of amino acids due to mutations, which play a role in the activation of the trypsinogen precursor or the activated trypsin, was reported to result in pancreatitis [23]. Moreover, the substitution of alanine to valine at position 16 (A16V) was reported to result in the abnormal processing of the trypsinogen precursor; whereas mutations of the D16A, D22G, and N29I, or N29T caused abnormalities in the activation of trypsin [24]. Mutations in the R122H or R122C have been known to interfere with the inactivation of activated trypsin [24]. Recurrent pancreatitis with severe abdominal pain could be refractory to conventional non-steroidal anti-inflammatory drugs and could interfere with social activities; moreover, severe cases are subjected to surgical resection of the inflamed regions [19]. Inflammation with infiltration of lymphocytes and neutrophils can damage cells and lead to malignant transformation, and the European study indicated an increasingly high risk of pancreatic cancer unrelated to the genotype after the age of 50 years [19]. Moreover, the correlation of pancreatic duct inflammation with epithelial destruction, regeneration, and cellular transformation remains to be understood perfectly. Therefore, the full study on HP-specific PSCs derived from patients would be needed to elucidate the mechanism and for drug screening.

Several causative genes of HP have been reported [18–20], but the sequencing study indicated that mutation in *PRSS1* gene is one of the most common causes of HP [22]. In this study, we established ESCs harboring *PRSS1* mutation and successfully performed the BC method to reproduce the HP phenotype in mice.

## RESULTS

### Establishment of a disease-specific PSC model using mouse ESCs

To confirm critical genes mutated in HP, we checked the mutated genes, the major mutation residues, and the mutation rates in HP. The result showed that Prss1 was the most frequently mutated gene in HP (Table 1). Most frequent mutation of *PRSS1* is R122H and transgenic mouse models with R122H-mutant Prss1 have been reported [25, 26]. On the other hand, the N29I mutation was the second most frequent and induces exocrine pancreatic insufficiency earlier than other mutations [23, 27]. These reports suggest that N29I mutation is a critical cause in HP. However, the precise mechanism for disease development remains to be elucidated. Moreover, a mouse model with N29I-mutant *PRSS1* is absent. Thus, it is important to establish the model reproducing HP with N29I mutation in Prss1.

**Table 1:** List of causative genes of HP

Gene	Major Mutation	Mutation Rate	Reference
PRSS1	N29I, R122H	80%	[35], [36]
SPINK1	N34S	20%	[35]
CFTR	F508del, 5T allele	30%	[36, 37]

**Figure 1:**
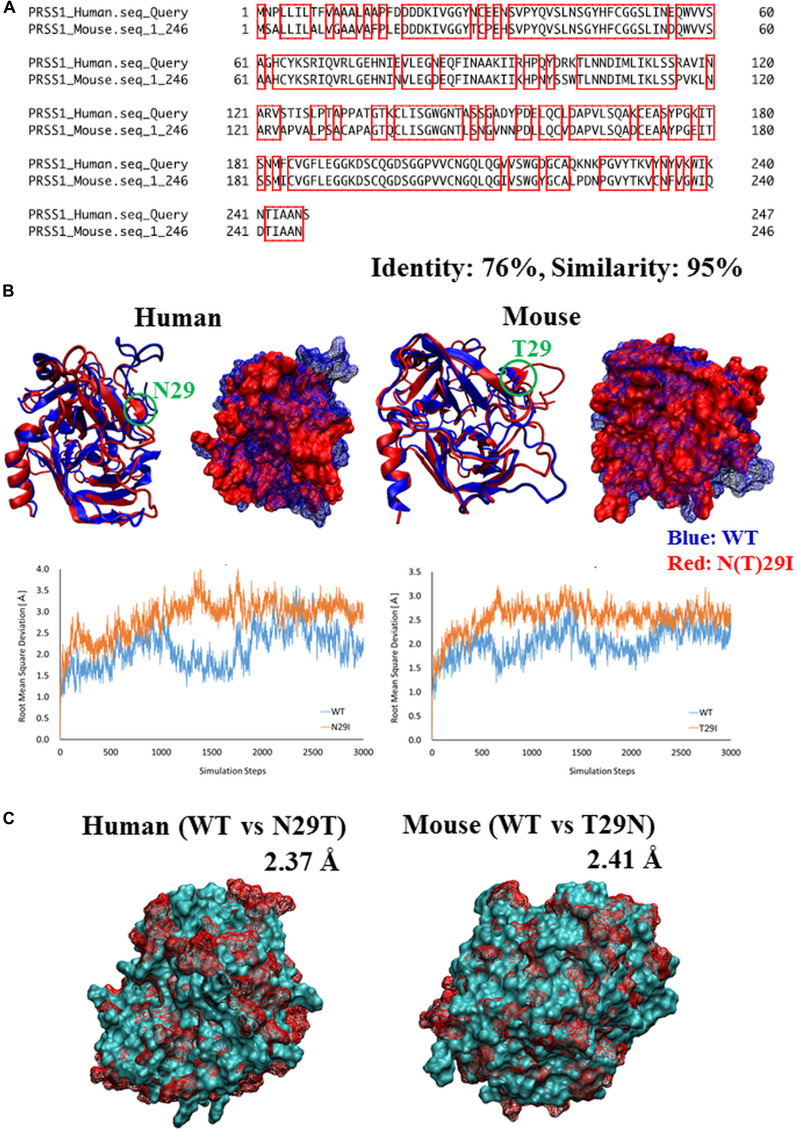
Comparison of Prss1 between human and mouse **(A)** Comparison of sequences of *PRSS1* between human and mouse. The regions surrounded by red line are common residues between human and mouse. **(B)** Comparison of 3D structure of Prss1 between WT and N29T (human) or T29N (mouse). The difference between the two structures is represented by root mean square deviation (RMSD). **(C)** Comparison of 3D structure of Prss1 between WT and N (T)29I in human and mouse. Comparison of flexibility between WT and N (T)29I in the most flexible region (human: residues 146-156, mouse: residues 187-200) by thermal vibration. Vertical axis: RMSD, Horizontal axis: simulation steps.

**Figure 2:**
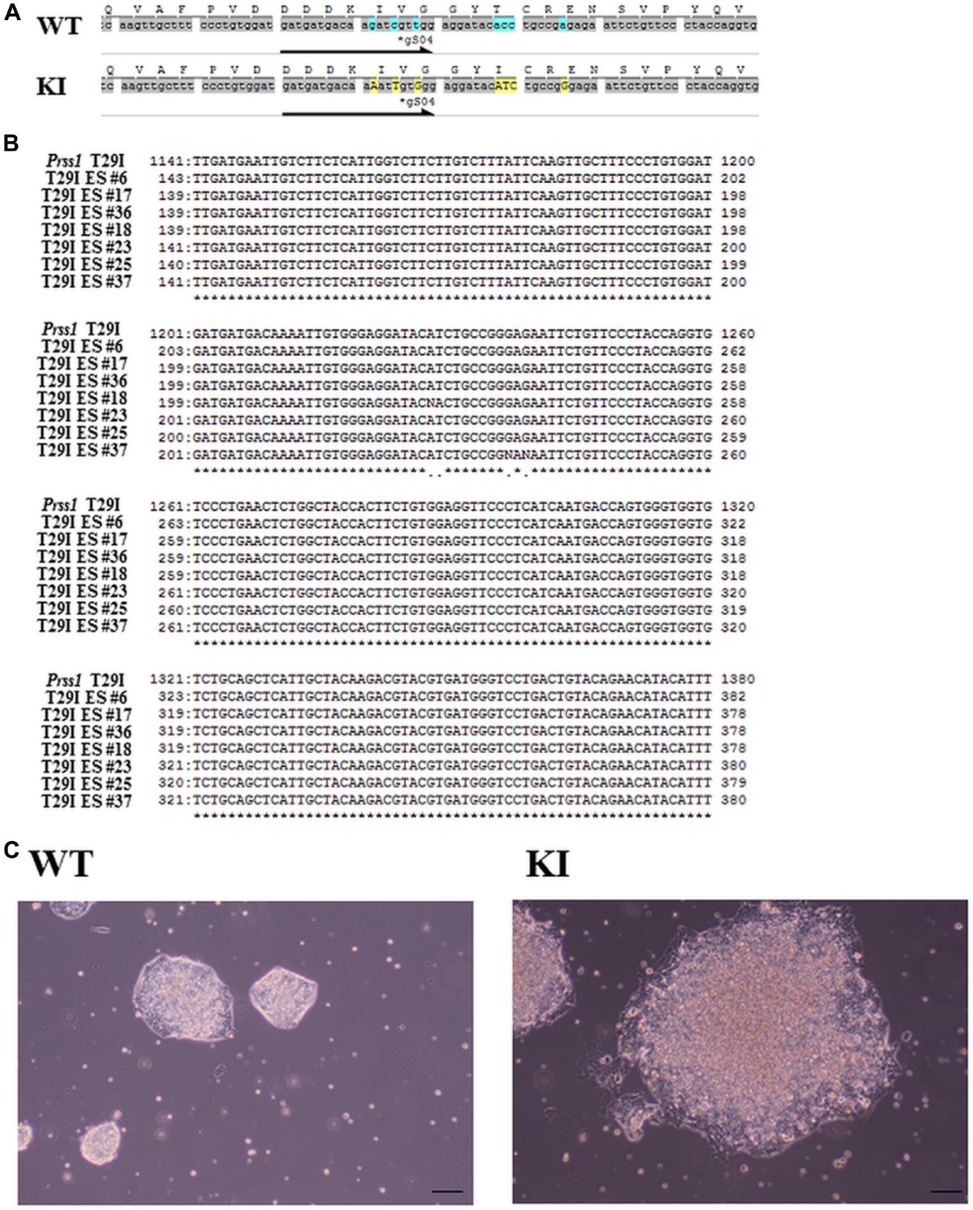
Generation of Prss1^T29I^ ESC **(A)** The sequence of *Prss1* in wild-type mice ESCs and Prss1^T29I^ ESCs. **(B)** The sequence of mouse ES cells after the introduction of mutation. **(C)** Phase contrast microscope images of ESCs. The Wt on the left side and the PRSS1^T29I^ on the right ES indicate the colony formation of undifferentiated states in the medium with LIF. Scale bar, 200 µm. *PRSS1*, the cationic trypsinogen or protease serine 1; Wt, wild type; Prss1^T29I^ ESC; Embryonal stem cells with CRISPR/CAS9-mediated mutations of T29I in *Prss1* gene; LIF, leukemia inhibitory factor.

To establish the disease-specific PSCs that correspond to HP in an animal model, we also compared the *PRSS1* genes in mice and humans and found 95% similarity and 76% identical amino acid sequences (Figure 1A), indicating conservation of the peptide sequence of the Prss1 protein among the species. Given that the 29th amino acid residue of Prss1, which causes HP, was different between human (H) and mouse (T), we created both Prss1 structures using the homology modeling method, performed molecular dynamics (MD) simulation to investigate for changes of flexibility after mutation of the 29th amino acid residue, and found that each structure was destabilized by the mutation in the 29th amino acid residue (Figure 1B). Moreover, given that the phenotype of HP might depend on the original residue if the human Prss1 mutation is mimicked in a mouse model, we created two three-dimensional (3D) structures by exchanging the 29th residue in the Prss1 of human and mouse and comparing these with the structure of each Prss1, and confirmed that none of the residues affected the original protein structure, suggesting that changing the structure by mutating to isoleucine did not depend on the original residue (Figure 1C). Here we decided to introduce T29I mutation into mouse ESCs and synthesized the gRNA (Figure 2A). By introduction of the gRNA into mouse ESCs and dsDNA with the mutated sequence (T29I), we established Prss1-mutant (Prss1^T29I^) ESCs. After the colonies were grown, the mutated sequence in the ESCs was checked by target DNA-seq (Figure 2B). The Prss1^T29I^ ESCs and wild-type ESCs were cultured and maintained successfully in an undifferentiated state in a medium that contained LIF (Figure 2C).

**Figure 3:**
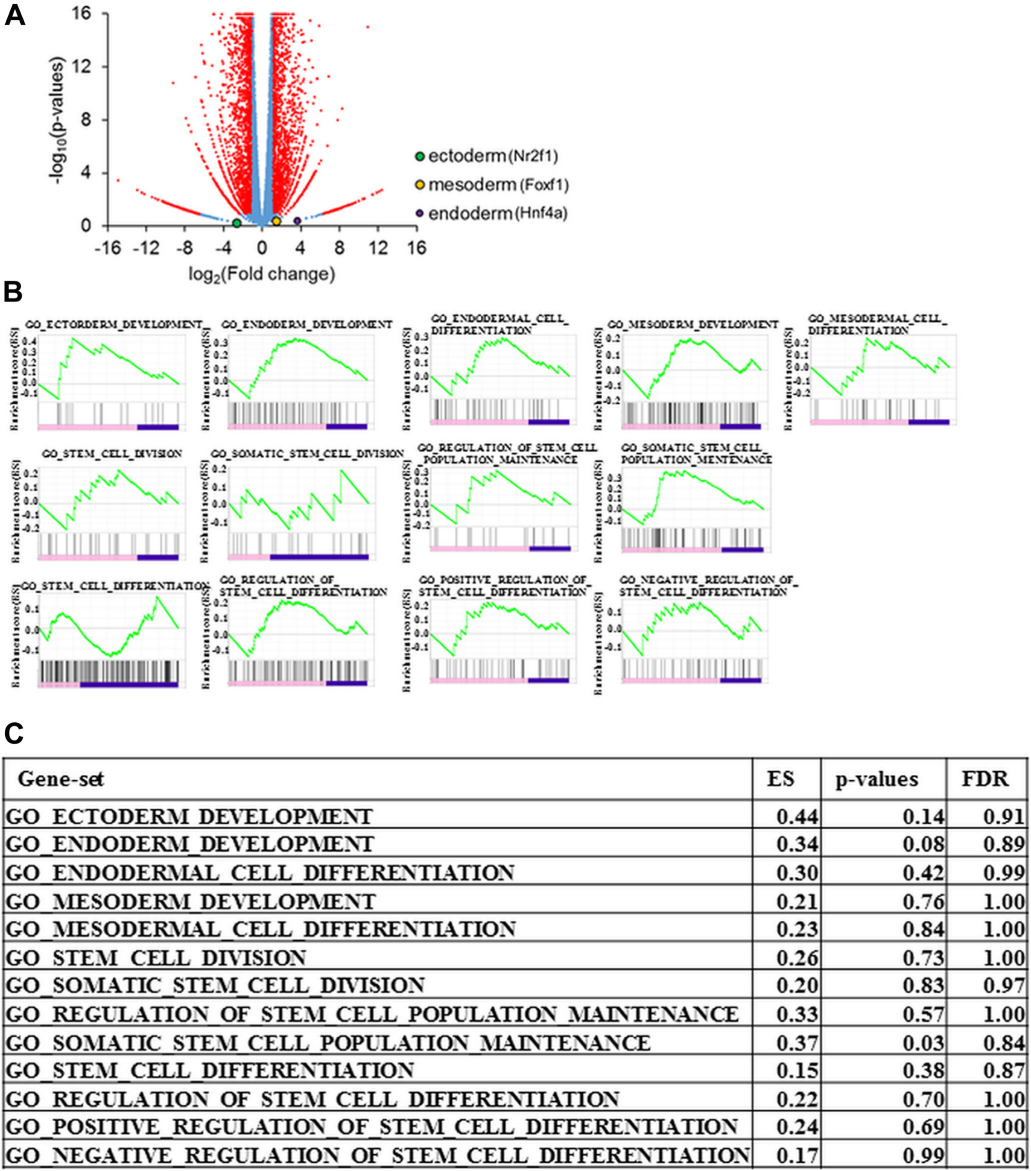
Gene expression of Prss1^T29I^ ESCs **(A)** Volcano plot for the gene expression of Prss1^T29I^ against wild-type ESCs. Horizontal axis: logarithm of fold change in the gene expression for Prss1^T29I^ against wild-type ESCs. Vertical axis: logarithm of p-values. **(B)** Enrichment plot of the gene sets involved in differentiation. **(C)** Enrichment Score (ES), p-values, and false discovery rate (FDR) of the gene sets involved in differentiation.

**Figure 4:**
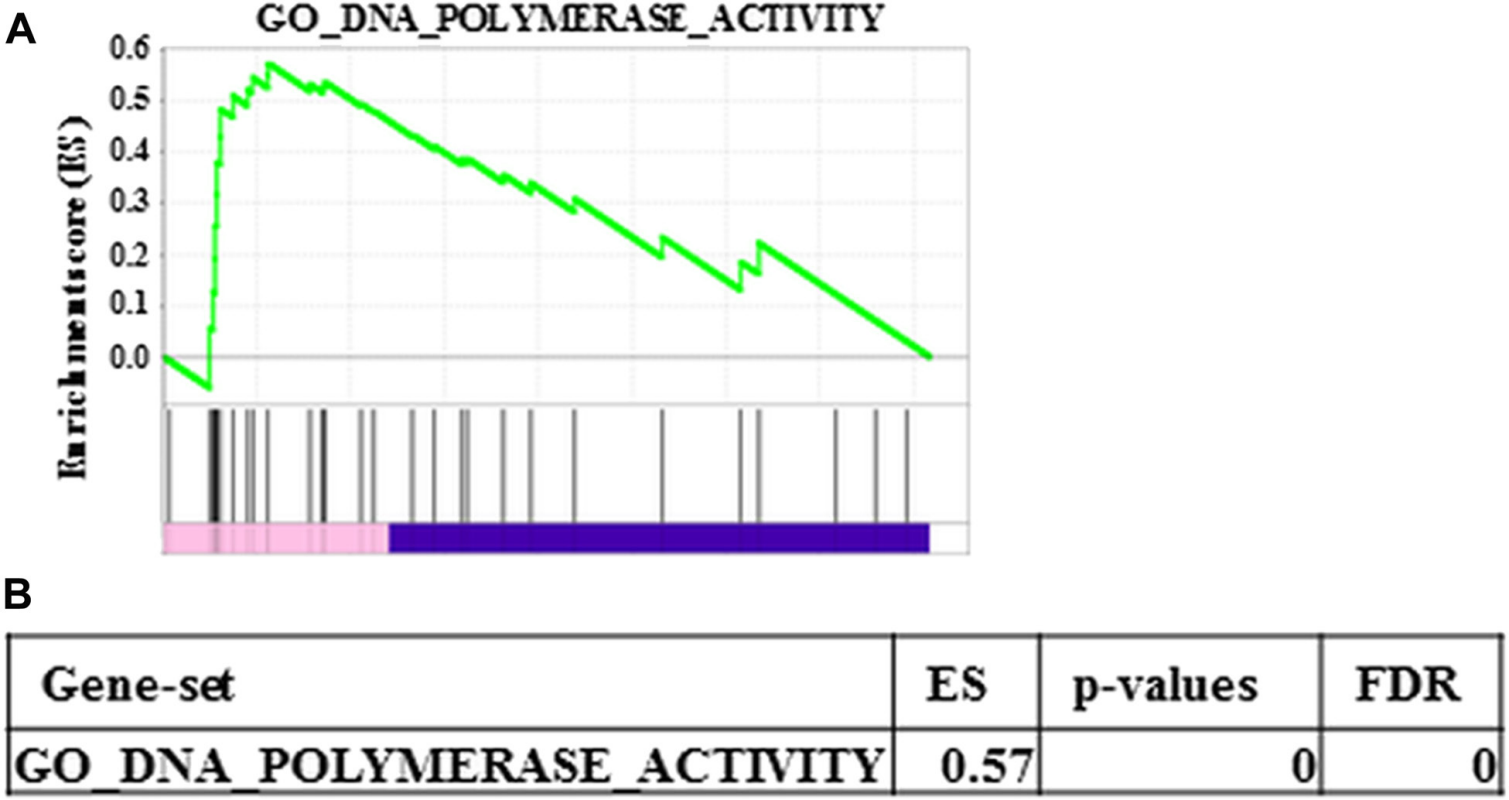
Most enriched gene set between wild-type ESCs and Prss1^T29I^ **(A)** Enrichment plot of the most enriched gene set between wild- type ESCs and PRSS1^T29I^. **(B)** ES, p-values and FDR of the most enriched gene set between wild-type ESCs and PRSS1^T29I^.

Prss1^T29I^ ESCs were established by CRISPR/CAS9 technology but needed to maintain chimera-forming ability equivalent to wild-type ESCs to reproduce HP by the BC method. To check this, first, the expression of *PRSS1* in each tissue was examined using BioGPS to investigate the influence of Prss1 mutation on the whole body. As a result, *PRSS1* was expressed in the pancreas alone in both human and mouse (Supplementary Figure 1A, 1B), indicating that the Prss1 mutation would affect only the pancreas. Second, the gene expression profiles were compared between ESCs to evaluate the chimera-forming ability of Prss1^T29I^ ESCs. As a result, the expression of each differentiation marker was similar between Prss1^T29I^ ESCs and wild-type ESCs (Figure 3A). Furthermore, the GSEA showed that the gene sets involved in both differentiation and stem cells were not enriched (Figure 3B, 3C). The most enriched gene set between Prss1^T29I^ and wild-type ESCs was the set involved in DNA polymerase (Figure 4A, 4B). The use of CRISPR/CAS9 technology might have affected DNA synthesis, but an enrichment score of 0.57 was not much. Therefore, the Prss1^T29I^ would retain its chimera-forming ability.

### Reproduction of HP using disease-specific PSCs

**Figure 5:**
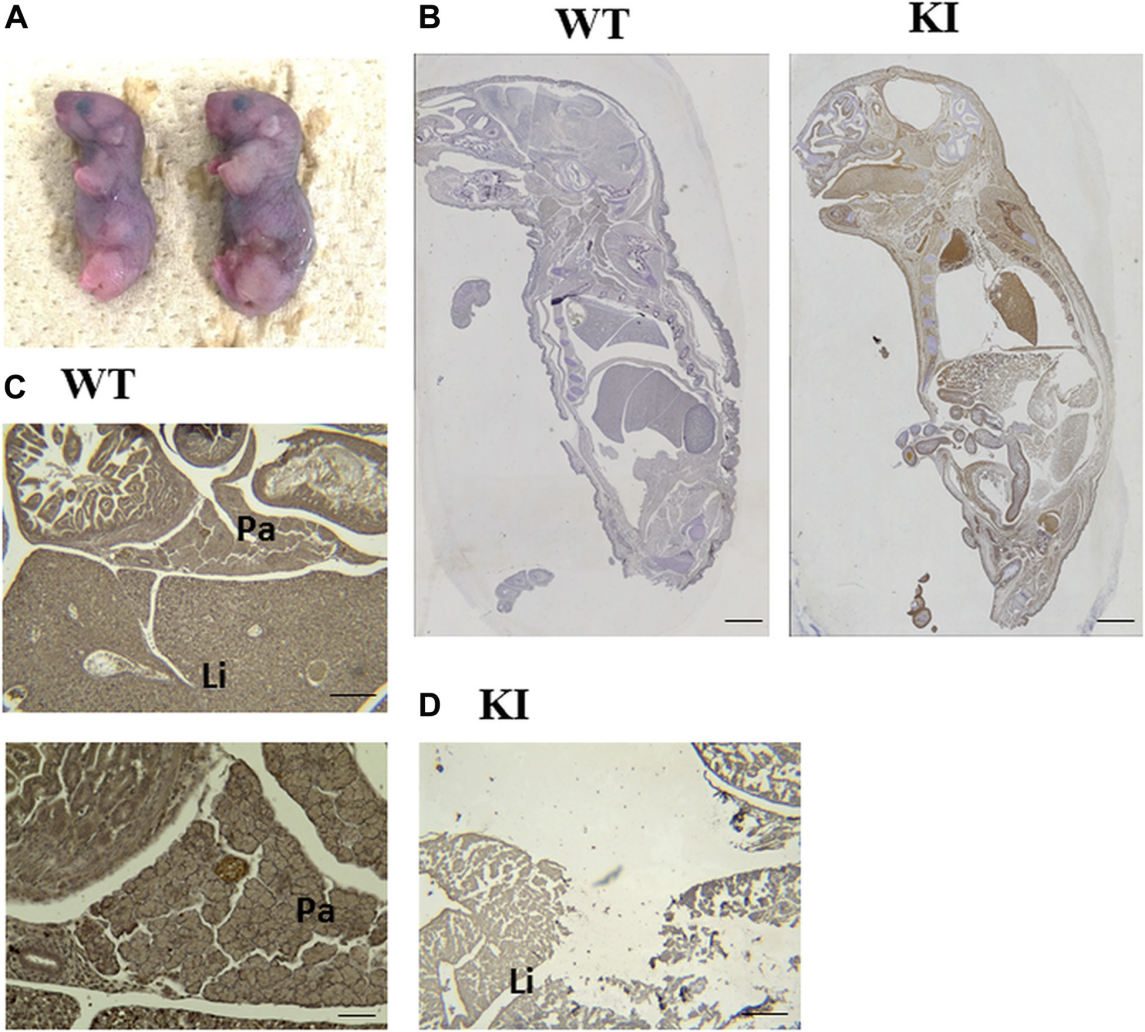
Reproduction of HP **(A)** Image of newborn mice with Prss1^T29I^. **(B)** Anti-Gfp antibody immunostaining experiment on E19.5-day embryos. The tissue derived from Prss1^T29I^ ESCs are Gfp-positive, which is shown as a brown chromogen color. Scale bar, 200 µm. **(C, D)** Anti-Ptf1a antibody immunostaining experiment on E19.5-day embryos. (C) The pancreas tissues of wild-type mice are Ptf1a-positive, which is shown as a brown chromogen color at E19.5. Scale bar, above: 50 µm, below: 20 µm. (D) The pancreas of mice with Prss1^T29I^ couldn’t be detected at E19.5. Scale bar, 50 µm. KI; mice knocked-in Prss1^T29I^, Pa; Pancreas, Li; Liver

The *Pdx1*-null mice that lacked pancreas were dead at two or three days after birth. However, introduction of wild-type ESCs in *Pdz1*-deficient blastocysts was reported to result in the complete rescue of the lethal phenotypes that harbored pancreatic tissues, with 100% contribution of wild-type ESCs [14]. In the field of regenerative medicine, the BC methods have emerged recently organ formation. Here we applied the BC method for the contribution of disease-specific Prss1^T29I^ ESCs and the causative formation of pancreatitis. In this experiment, we injected Prss1^T29I^ ESCs into the blastocysts that were derived from *Pdx1*-null mice and were transferred to another female, in which pregnancy was mimicked by injection of hCG, creating a provisional belly. Although twelve mice which were chimera with Prss1 mutated ES cells were born, all chimeric mice died by 1.5 days after birth (Figure 5A).

**Figure 6:**
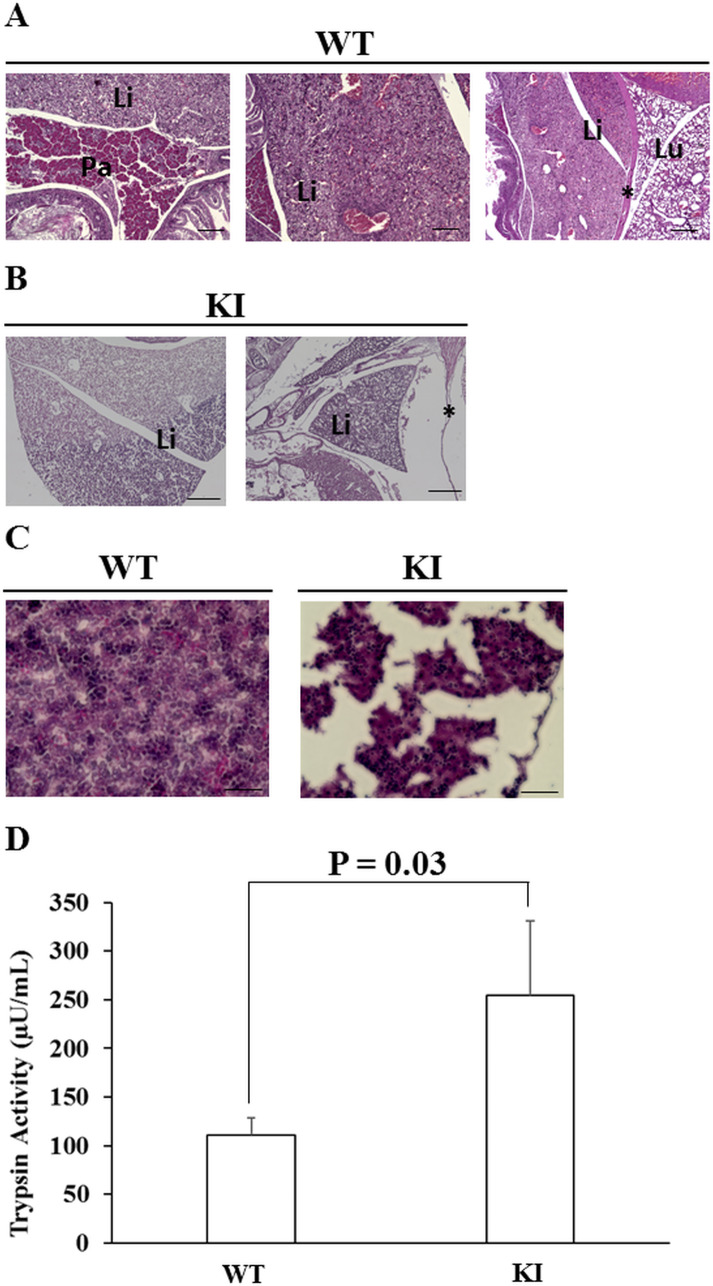
Trypsin activity of the HP model **(A)** The hematoxylin and eosin (HE) staining of wild-type mice at E19.5. Scale bar, 20 µm. **(B)** The HE staining of mice with Prss1^T29I^ at E19.5. Scale bar, 50 µm. **(C)** The HE staining of the liver of the wild-type mice and the mice with Prss1^T29I^ at E19.5. Scale bar, 30 µm. **(D)** The results of the measurement of trypsin activity. Each value represents the mean ± S.D. (n = 3). KI; mice knocked-in Prss1^T29I^, Pa; Pancreas, Li; Liver, Lu; Lung, ^*^; Diaphragm.

To confirmed whether the Prss1^T29I^ ESCs contributed to the tissues of the whole body and pancreas, we performed an anti-Gfp antibody immunostaining experiment on E19.5-day embryos, because the Prss1^T29I^ ESCs were labeled by the Gfp expression. The results indicated that Gfp-positive cells were detected in the whole body (Figure 5B). These suggested that the injected Prss1^T29I^ ESCs contributed to the formation of chimera. However, the pancreas in mice with Prss1^T29I^ was unclear. Thus, we performed an anti-Ptf1a, a pancreas marker, antibody immunostaining on E19.5-day embryos. As a result, the wild-type mice had formed a complete pancreas and the pancreatic islet was strongly stained, whereas pancreatic organ of the mice with Prss1^T29I^ was undetectable even anti-Ptf1a antibody immune-staining (Figure 5C, 5D). Moreover, the liver of mice with Prss1^T29I^ showed severely destroyed structures and thin diaphragm (Figure 6A–6C), presumably due to the effects of activated trypsin. We then measured the trypsin activity of mice with Prss1^T29I^ and wild-type mice. The results indicated that the trypsin activity of mice with Prss1^T29I^ was significantly higher than wild-type mice (Figure 6D). The present study showed that the BC methods with Prss1^T29I^ ESCs can reproduce the phenotype of HP in a model.

## DISCUSSION

Although the BC method using disease-specific PSCs would be promising to reproduce hereditary diseases, there is no report to reproduce human diseases using BC method. In the present study, thus, we investigated to reproduce HP using the BC method.

A recent study indicated that the BC method was applicable to intercross species of mice ES-in-rats blastocysts as well as rats ES-in-mice blastocysts [14]; also, it has been demonstrated that swine ES-in-swine blastocysts [17], as well as mice ES-in-rats blastocysts were useful to maintain the insulin level in glucose metabolism, as experimental models [28]. Very importantly, these reports suggest that human diseases could be reproduced in other animals by BC method. In conventional researches for disease therapy, disease model animals based on treatment of a reagent or animal genetic modification have been used [29]. However, even though these methods could mimic the phenotype of human disease, it is difficult to reproduce the causing mechanism of human diseases for the following reasons; 1) not only target tissues but also non-target tissues are affected by the treatment, 2) only phenomena in animal tissues (not humans) could be investigated. In this regard, BC method can reproduce tissues of intercross species in only target organs. Therefore, BC method would be promising to elucidate mechanisms of human disease and can contribute to the progression of medicine.

Because *PRSS1* was the most frequently mutated gene in HP, we established the corresponding animal model. The sequence study indicated that asparagine was substituted to isoleucine at position 29 (N29I) in human, although a previous study indicated that a transgenic mouse that overexpressed the amino acid substitution of threonine to isoleucine at position 29 (T29I) showed the phenotype of recurrent acute and chronic pancreatitis [23], through the involvement of apoptosis and necrosis of pancreatic cells [30–33]. Furthermore, overexpression of human N29I in mouse cells resulted in the induction of apoptosis of murine acinar cells, which is of a similar phenotype with that of pancreatitis [33]. These data indicated that N29I in human and T29I in mouse have similar effects on the onset of pancreatitis. Similarly, the present study indicated that the computational predicted structure of T29I was similar with that of N29I. In this study, we used the CRISPR/CAS9-mediated mutation of T29I substitution in the endogenous copy of the *Prss1* gene allele in a mouse BC model, suggesting that T29I was a proper counterpart of human Prss1^N29I^ mutation.

Based on the structural study and GSEA analysis, we proposed that the injection of Prss1^T29I^ did not affect the undifferentiated state and chimera-formation ability of ESCs. Nevertheless, we have to consider the possibility that mice with Prss1^T29I^ mutation might express a more severe phenotype than human Prss1^N29I^, based on the fact that the mice died immediately after birth. It has reported that *Pdx1* KO mice are born alive but die within a week after birth, presumably due to a complete absence of pancreatic tissues and malformations. In addition, *Pdx1* KO mice have no trypsin activity, since acinar cells in *Pdx1* KO mice are absent [14]. The phenotype of Prss1^T29I^ mice is significantly different from that of *Pdx1* KO mice. It has been reported that the N29I mutation of Prss1 induces exocrine pancreatic insufficiency earlier than other mutations [27]. Our results in this study might reflect early outcome in human HP, though further improvement of the BC method is necessary to elucidate disease pathophysiology. The development of the BC method makes it possible to reproduce human diseases in other organs, diseases and species, and it will be able to construct human organs in animals such as pigs and to apply to disease research and drug screening.

**Figure 7:**
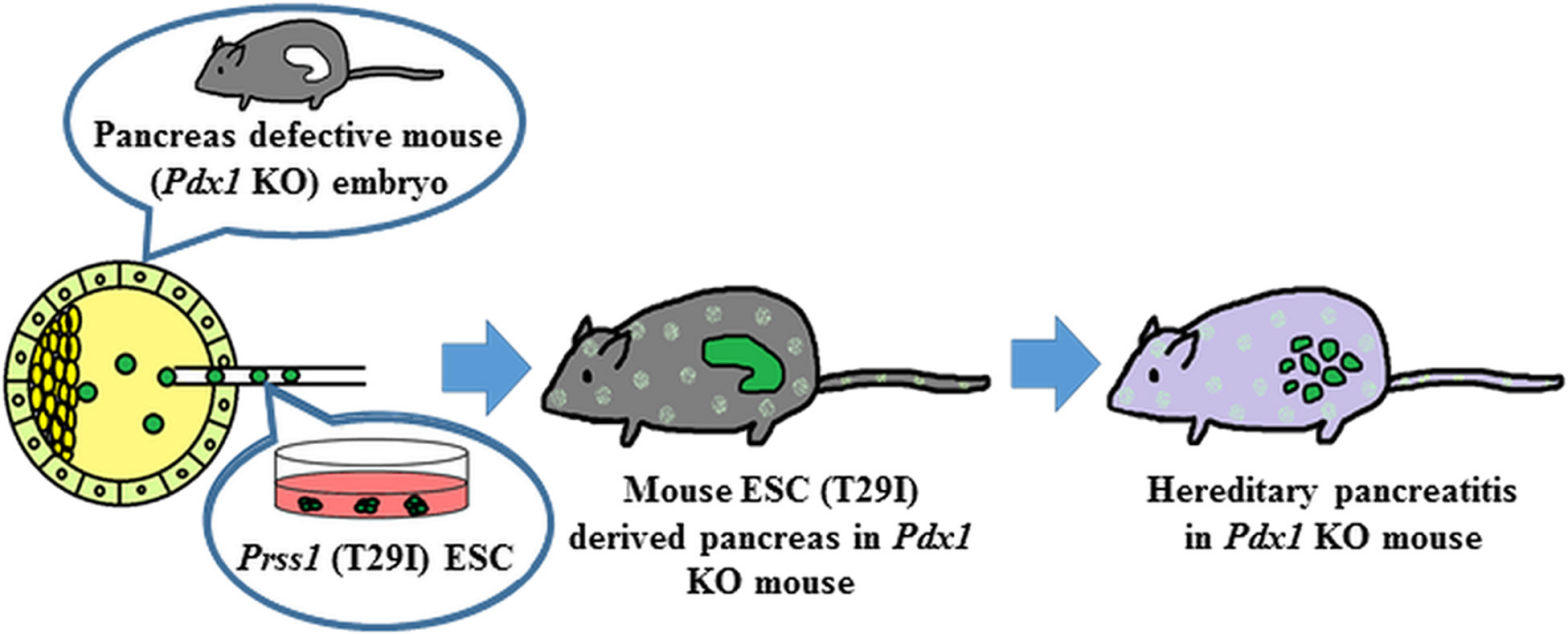
Reproduction of HP by BC method Mouse ES cells harboring mutations in the Prss1 were injected into the blastocysts with deficient *Pdx1* gene. The blastocysts injected into the Prss1-mutant ES cells induced trypsin activation like human HP.

In conclusion, we applied to the BC method with Prss1-mutant ESCs to reproduce HP in mouse (Figure 7). The embryos derived from Prss1-mutant ESCs showed trypsin activation and autolysis, mimic to human HP. As the best of our knowledge, the present study is the first report for reproducing human disease by utilizing BC method. Although the application of this method to human cells has ethical issues to be overcome, utilizing BC method would be an attractive tool for the study of human diseases and can contribute to the drug discovery and regenerative medicine.

## MATERIALS AND METHODS

### ESCs culture

EGR-G101 cells, which were derived from a male mouse embryo, were cultured in Dulbecco's modified eagle medium (Nacalai Tesque, Kyoto, Japan) containing 15% FBS (Thermofisher, Tokyo, Japan); 1% non-essential amino acids (Nacalai Tesque); 1% sodium pyruvate (Nacalai Tesque); and LIF (Nacalai Tesque) at 37°C in a humidified atmosphere with 5% CO_2_.

### CRISPR editing

The ES cells were transfected gRNAs and pX330 vector using lipofectamine 3000 (Life Science Technologies, Tokyo, Japan). gRNA was following sequence. 5′- gatgatgacaagatcgttgg -3′. The double strand break was repaired by dsDNA with the mutated sequence (T29I). The cells were cultured on 369 well plates in a single cell per each well. The cells were selected with 1 µg/µL puromycin. After the colonies were grown, we checked the *Prss1* DNA sequence using 310 Genetic Analyzer (PerkinElmer, Tokyo, Japan). *Prss1* sequence primer was the following sequence. 5′ - ggacatactgccacatacct -3′.

### Embryo preparation

Preparation of *Pdx1* knockout (KO) mouse embryos was carried out according to Nagy et al., 2003 [34]. In brief, mouse 8-cell/morula stage embryos were collected in Medium 2 (Millipore, Tokyo, Japan) from oviduct and uterus of mice E2.5. These embryos were cultured into potassium simplex optimized medium with amino acids (Millipore) and were cultured for 24 hours for blastocyst injection.

### Establishment of a disease-specific PSCs model using mouse embryonic stem cells

The animal study was conducted in accordance with the guidelines of the Institutional Review Board for the Care of Animal Subjects at Osaka University (approval No. 30-011-011, chaired by professor Y. Kaneda). Prss1^T29I^ ESCs were generated using gRNAs in the CRISPR/CAS9 System. On the first day, pregnant mare serum gonadotropin (5 IU) was administered intraperitoneally to female *Pdx1* KO hetero C57BL/6J mice. On the third day, human chorionic gonadotropin (hCG), 5 IU was administered intraperitoneally to the mice. Subsequently, the mice were mated with male *Pdx1* KO hetero C57BL/6J mice. On the sixth day, the blastocysts were collected from the female mice and were injected with mutated Prss1 ESCs. Subsequently, the blastocysts were cultured overnight. On the seventh day, the cultured blastocysts were transplanted into the uteri of the foster mothers.

### RNA-seq for ESCs

RNA was extracted from each ESCs and was sequenced by Hi-seq2500. Gene expression was analyzed using CLC Genomics Workbench Ver.9.0 and GSEA software. RNA-seq data were mapped with the following parameters: 1) maximum number of allowed mismatches was 2; 2) minimum length and similarity fraction was set at 0.8; and 3) maximum number of hits per read was 10. The gene set enrichment analysis (GSEA) was performed using c5. all. v6.0. symbol. gmt in Gene Sets database.

### Immunostaining

Immunohistochemical analysis was performed on 3.5-μm, paraffin-embedded sections from the chimera mice. The paraffin-embedded sections were de-paraffinized in Hemo-De (Farma, Japan) and were rehydrated in a graded series of ethanol. The slides were heated in an antigen retrieval buffer for 40 minutes, blocked with goat or horse serum for 20 minutes at room temperature, and incubated with monoclonal mouse anti-Gfp antigen antibody (1:1000, Abcam, England) or monoclonal mouse anti-PTF1A antigen antibody (1:50, BD Biosciences, US) overnight at 4°C. The Vectastain ABC System (Vectastain, Funakoshi, Japan) was used to visualize the antigens. Counter-staining was performed using hematoxylin only or HE.

### Trypsin activity assay

The abdomen of each mouse at birth (day 0) was cut and homogenized in PBS on ice. They were centrifuged and collected the supernatant as a sample. The trypsin activity of samples was evaluated using a Trypsin Activity Assay Kit (ab102531, Abcam, San Francisco, CA), according to the manufacture’s protocol. These measurements were performed three times independently.

### Protein structure analysis

We created the 3D structures of wild-type (WT) Prss1 and mutated Prss1 (human: N29I, mouse: T29I) using the homology modeling method, based on the crystal structure of the human cationic trypsin G193R mutant (PDB ID: 4WWY). Then, the structures of the WT and mutated type were subjected to energy minimization in the water phase of the AMBER force field using the AMBER12 program package. Using these minimized structures, we performed molecular dynamics (MD) simulation to investigate the differences in flexibility between the WT and mutated structures. We performed the MD simulation (i. e., elevated temperature process and thermodynamically conformational sampling) at around 37.0°C (310 K) using the periodic boundary condition. The flexibility of each protein structure was compared by calculating the root mean square deviation (RMSD) from each initial coordinate. We created the structures that were exchanged at the 29th amino acid residue in Prss1 between humans (N→T) and mice (T→N) and compared the original structures with the mutated structures. The RMSD was calculated as an index of structural difference in each minimized structure after *in silico* annealing processes.

### Statistical analysis

Prior to the significance test, the distribution of data was confirmed using an F-test. Statistically significant differences were determined by the Student’s t test.

## SUPPLEMENTARY MATERIALS FIGURES AND TABLE


