# Supplementary material for: Hereditary pancreatitis model by blastocyst complementation in mouse
Source: Oncotarget. 2020 Jun 2;11(22):2061–73. doi: 10.18632/oncotarget.27595 (PMC7275788; doi:10.18632/oncotarget.27595)
Supplement: Supplementary file 1 [file oncotarget-11-2061-s001.pdf]

Hereditary pancreatitis model by blastocyst complementation in mouse

SUPPLEMENTARY MATERIALS

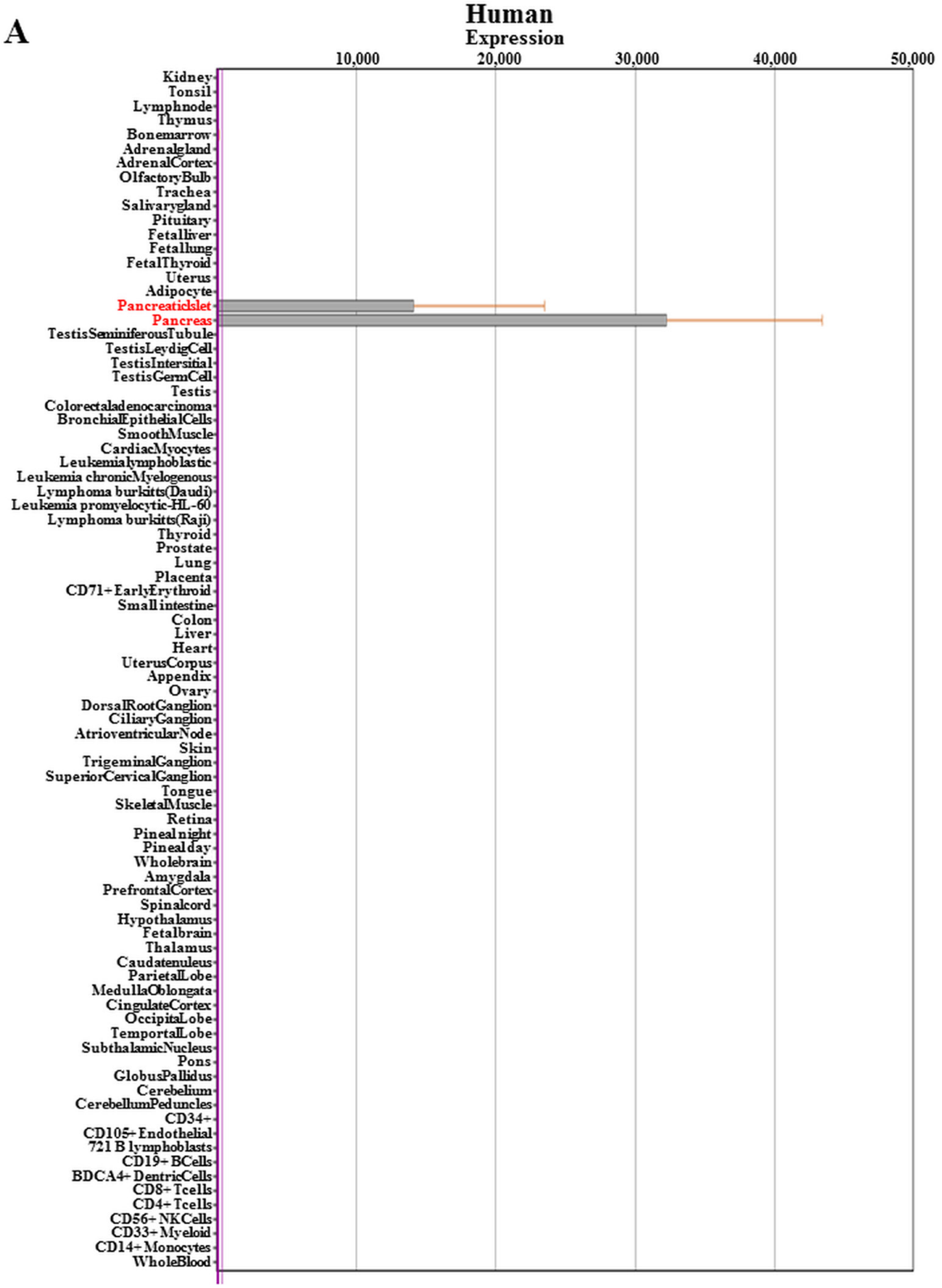

Supplementary Figure 1A: *PRSS1 expression* in whole body of **(A)** human. Horizontal axis: Expression values. Vertical axis: Tissues.

B

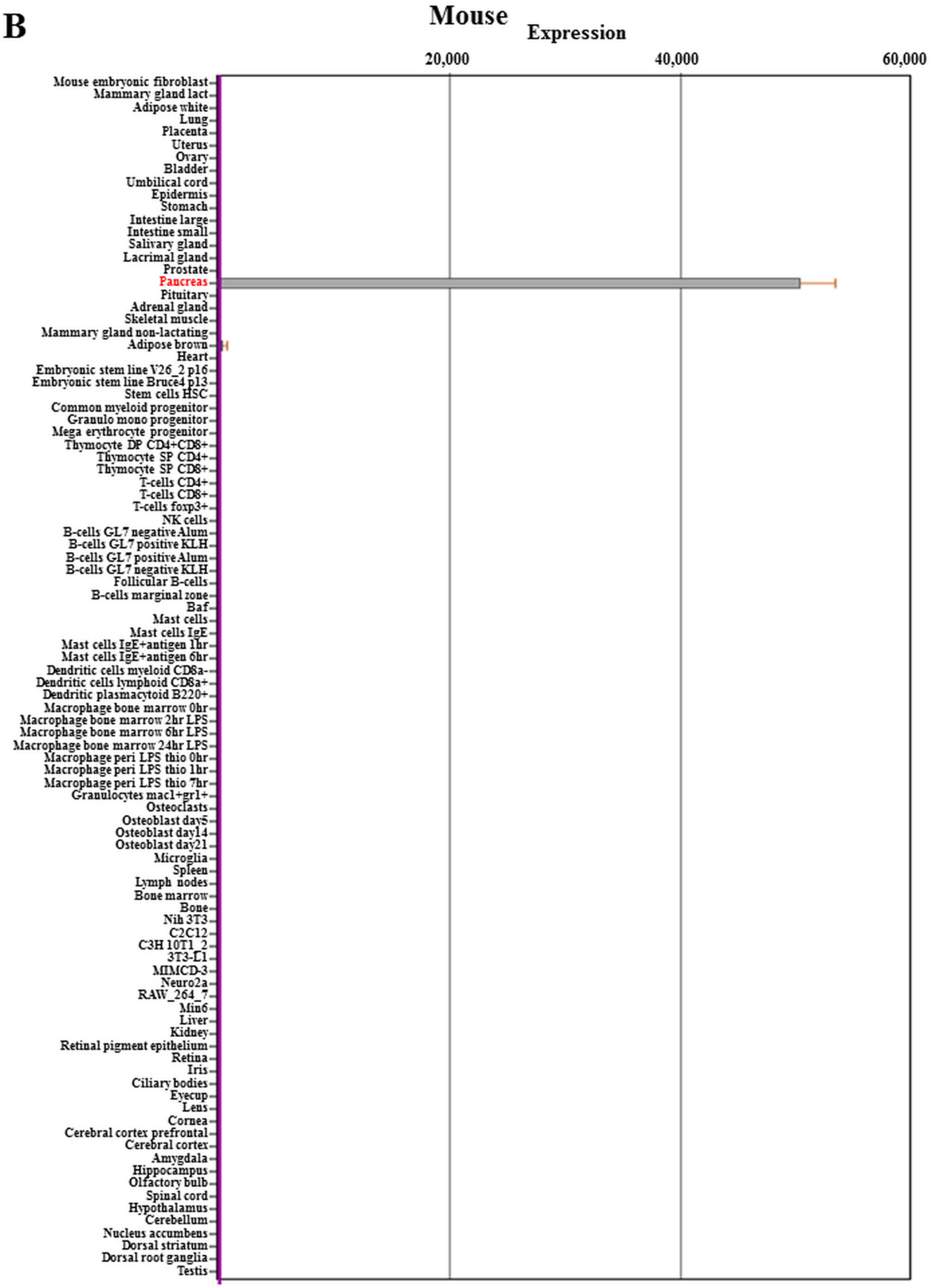

Supplementary Figure 1B: *PRSS1 expression* in whole body of **(B)** mouse. Horizontal axis: Expression values. Vertical axis: Tissues.

Supplementary Table 1: The descriptions of GO library

Gene-set	Description
GO_ECTODERM_DEVELOPMENT	The process whose specific outcome is the progression of the ectoderm over time, from its formation to the mature structure. In animal embryos, the ectoderm is the outer germ layer of the embryo, formed during gastrulation. [GOC:dph, GOC:tb]
GO_ENDODERM_DEVELOPMENT	The process whose specific outcome is the progression of the endoderm over time, from its formation to the mature structure. The endoderm is the innermost germ layer that develops into the gastrointestinal tract, the lungs and associated tissues. [GOC:dph, GOC:tb]
GO_ENDODERMAL_CELL_ DIFFERENTIATION	Any process that modulates the frequency, rate or extent of endodermal cell differentiation. [GO_REF:0000058, GOC:als, GOC:TermGenie, PMID:23154389]
GO_MESODERM_DEVELOPMENT	The process whose specific outcome is the progression of the mesoderm over time, from its formation to the mature structure. The mesoderm is the middle germ layer that develops into muscle, bone, cartilage, blood and connective tissue. [GOC:dph, GOC:tb]
GO_MESODERMAL_CELL_ DIFFERENTIATION	The process in which a relatively unspecialized cell acquires the specialized features of a mesoderm cell. [GOC:dgh]
GO_STEM_CELL_DIVISION	The self-renewing division of a stem cell. A stem cell is an undifferentiated cell, in the embryo or adult, that can undergo unlimited division and give rise to one or several different cell types. [GOC:jid, ISBN:0582227089]
GO_SOMATIC_STEM_CELL_ DIVISION	The self-renewing division of a somatic stem cell, a stem cell that can give rise to cell types of the body other than those of the germ-line. [GOC:jid, ISBN:0582227089]
GO_REGULATION_OF_STEM_CELL_ POPULATION_MAINTENANCE	Any process in by an organism or tissue maintains a population of neuronal stem cells. [CL:0000047, GOC:dos, GOC:yaf, PMID:11399758]
GO_SOMATIC_STEM_CELL_ POPULATION_MAINTENANCE	Any process by which an organism retains a population of somatic stem cells, undifferentiated cells in the embryo or adult which can undergo unlimited division and give rise to cell types of the body other than those of the germ-line. [GOC:bf, ISBN:0582227089]
GO_STEM_CELL_DIFFERENTIATION	The process in which a relatively unspecialized cell acquires specialized features of a stem cell. A stem cell is a cell that retains the ability to divide and proliferate throughout life to provide progenitor cells that can differentiate into specialized cells. [CL:0000034, GOC:isa_complete]
GO_REGULATION_OF_STEM_CELL_ DIFFERENTIATION	Any process that modulates the frequency, rate or extent of stem cell differentiation. [GOC:obol]
GO_POSITIVE_REGULATION_OF_ STEM_CELL_DIFFERENTIATION	Any process that activates or increases the frequency, rate or extent of stem cell differentiation. [GOC:obol]
GO_NEGATIVE_REGULATION_OF_ STEM_CELL_DIFFERENTIATION	Any process that activates or decreases the frequency, rate or extent of stem cell differentiation. [GOC:obol]
GO_DNA_POLYMERASE_ACTIVITY	Catalysis of the reaction: deoxynucleoside triphosphate + DNA(n) = diphosphate + DNA(n+1); the synthesis of DNA from deoxyribonucleotide triphosphates in the presence of a nucleic acid template and a 3′hydroxyl group. [EC:2.7.7.7, GOC:mah]
